# Global prevalence of sexual dysfunction in cardiovascular patients: a systematic review and meta-analysis

**DOI:** 10.1186/s13643-024-02525-0

**Published:** 2024-05-20

**Authors:** Arash Ziapour, Mohsen Kazeminia, Mohammad Rouzbahani, Saeedeh Bakhshi, Nafiseh Montazeri, Murat Yıldırım, Hani Tadbiri, Farideh Moradi, Parisa Janjani

**Affiliations:** 1 Cardiovascular Research Center, Health Institute, Imam-Ali Hospital, Kermanshah University of Medical Sciences, Kermanshah, Iran; 2 Student Research Committee, Kermanshah University of Medical Sciences, Kermanshah, Iran; 3 Department of Psychology, Faculty of Science and Letters, Agri Ibrahim Cecen University, Ağrı, Türkiye; 4 Graduate Studies and Research, Lebanese American University, Beirut, Lebanon; 5 Islamic Azad University, Tehran Medical Branch, Tehran, Iran

**Keywords:** Prevalence, Sexual dysfunction, Cardiovascular, Systematic review, Meta-analysis

## Abstract

**Background:**

Sexual dysfunction poses a significant challenge for patients with cardiac conditions. Concerning the prevalence of sexual disorders in cardiovascular patients, several seminal studies conducted in various regions of the world have provided diverse facts and figures pertaining to sexual dysfunction among cardiovascular patients. Therefore, the present study aimed to analyze, summarize, and integrate the findings of seminal studies on the effect of underlying factors and estimate the global rate of sexual disorders in cardiovascular patients.

**Methods:**

The present systematic review and meta-analysis included studies conducted in 2003–2023. To find the relevant published academic papers, SID, MagIran, PubMed, Scopus, Web of Science (WOS), and Google Scholar databases were searched for keywords using MeSH/Emtree until January 14, 2023. The GRADEpro software was used to evaluate the quality of evidence. The heterogeneity of studies was checked using the I2 index.

**Results:**

An initial number of 2122 studies were found in the first search. Following a precise screening process based on predefined inclusion criteria, a total of 17 studies were deemed suitable for inclusion in the meta-analysis. The global prevalence of sexual disorders in cardiovascular patients was estimated at 62.6% (95% *CI*: 49.8–73.8%). As the results of the meta-regression showed that as the sample size increased, there was a significant decrease in the overall prevalence of sexual disorders among cardiovascular patients. Additionally, as the study years progressed, both age and JBI score exhibited an upward trend.

**Conclusion:**

The present findings showed a high prevalence of sexual disorders among cardiovascular patients. Therefore, it is recommended that experts and health policymakers enhance their focus on effectively preventing and controlling these disorders. Besides the evidence achieved very low certainty, it is important for the treatment team to prioritize the sexual relations of cardiovascular patients and focus on improving their sexual function. This should be seen as an essential aspect of their overall recovery process.

## Background

Cardiovascular diseases (CVDs) are considered among the major causes of mortality, accounting for one-third of all mortalities worldwide [1, 2]. CVDs account for half of the annual mortalities in developing countries [2, 3]. CVDs include ischemic heart disease, myocardial infarction, heart failure, coronary artery disease, peripheral arterial disease, chronic hypertension, and a number of other cardiovascular conditions [4–6].

The incidence of CVDs has grown due to inappropriate eating habits, reduced physical activity, increased stress, and smoking [7]. Coronary artery disease can cause physical and mental problems and adversely affect the patient’s quality of life (QoL) [1, 8–10]. Also, CVDs can cause trouble in sexual activity by disrupting its physical and mental aspects [11]. Some patients with cardiovascular diseases such as myocardial infarction, coronary artery surgery, or ischemic heart disease face problems in their normal sexual activities [12–14]. The prevalence of sexual dysfunction in patients with CVDs has been estimated at up to 89% [2, 15]. Another study also showed that patients with CVDs experience three times the rate of sexual dysfunction compared to individuals without any health issues [13]. The causes of sexual dysfunction in patients with CVDs include physical/psychological changes as well as side effects from medication usagE [1, 2]. Sexual dysfunction includes a combination of disorders in one’s ability to respond sexually to the partner [16, 17]. According to the European Society of Urology, sexual dysfunction (SD) is defined as a persistent inability to achieve and maintain a satisfactory erection for sexual intercourse [18]. Specific instances of sexual dysfunction include disorders such as libido disorder, male erectile dysfunction (ED), female sexual arousal disorder, premature ejaculation, male and female orgasm disorder, and sexual pain such as vaginismus and dyspareunia [17, 19]. The physical and mental problems caused by CVDs can disrupt sexual activity, leading to detrimental effects on patients’ quality, including reduced self-esteem and increased depression and a tendency self-isolation over time [20, 21]. Sexual dysfunction can predict CVDs before the onset of symptoms of a cardiovascular event, which indicates coronary artery disease [15]. Symptoms of SD precede clinical manifestations of CVDs by 3 to 5 years. This period of time may be ripe for early initiation of therapeutic measures for cardiovascular risk factors [20].

Epidemiological studies have revealed a high global prevalence of both SD and CVDs [1]. Many studies addressed this issue from multiple angles. Thompson et al. conducted pioneering research in 2005, linking SD with an increased risk of CVDs [22]. In their study involving 10,000 male participants, they showed that the severity of SD is associated with the risk of CVDs [22]. In another study, Rinkūnienė et al. showed that the overall prevalence of erectile dysfunction after myocardial infarction was 62% [23]. Furthermore, other scientific evidence has shown that ED sexual disorder is significantly more prevalent in patients with multiple risk factors for CVDs [24]. Additionally, several reviews have provided insights into the prevalence of sexual disorders [14, 25–27]. A substantial body of research has also shown that SD affects up to 42% of patients with CVDs [28, 29], while in general healthy statistics are different. For example, in the Polish population, 40% of women and 36.5% of men reported least one sexual dysfunction [30]. Also, in Iran, it was found that approximately 31% of women experience sexual dysfunction [31]. An old research shows that sexual disorders approximately 43% of women and 31% of men experience these problems [32]. By comparing the prevalence of sexual dysfunction in two healthy and cardiovascular disease groups, we understand that considering the abovementioned challenges and problems, more attention should be paid to sexual dysfunction in CVD patients [33]. It is evident that the prevalence of this disorder differs between men and women. For example, the results of a study showed that 37% of men and 80% of women with myocardial infarction had sexual problems [34]. Several other studies have shown the high risk of sexual dysfunction in both men and women with CVDs. Sexual dysfunction is more common in men than in women [1, 35]. Sexual dysfunction occurs in 75% of men with myocardial infarction (MI). Up to 57% of men is with implanted external defibrillator [13, 36], and such disorders are more common in men [2, 36]. The symptoms of sexual disorders in CVD patients are marked by a decrease or loss of sexual desire, avoidance of sexual activity, and sexual dissatisfaction [1]. The sexual problems among CVD patients have adverse consequences for both patients themselves and their partners [20].

In recent years, several seminal studies have been conducted worldwide to explore the prevalence of sexual disorders among patients with CVDs. Yet, the existing literature has investigated the prevalence in a limited geographical area with small sample sizes, and there is a lack of evidence into the potential effects of factors such as the year of study, sample size, and average age. Furthermore, there are also many differences in the prevalence rates reported in these studies. Thus, it is necessary to conduct a systematic review and meta-analysis to integrate, summarize, and resolve the contradictory findings. Also, it is necessary to investigate the effect of potential factors and also estimate the global prevalence of sexual disorders in cardiovascular patients. To the best of our knowledge, such a study has not been undertaken thus far. Therefore, the present study aimed to estimate the global prevalence of sexual disorders in cardiovascular patients through a systematic review and meta-analysis.

## Materials and methods

The present study was conducted as a systematic review and meta-analysis encompassing the years 2003 to 2023. This study was conducted based on PRISMA 2020 guidelines (http://www.prisma-statement.org/), which provided a comprehensive framework for conducting transparent and rigorous reviews. The current research followed a sequential process comprising the following distinct stages: Identification, screening, eligibility, and inclusion [37]. To reduce potential errors, inaccuracies, and publication bias, all the steps of searching, evaluating, identifying/selecting the studies, and extracting data were performed independently by two researchers. Any discrepancies or disagreements between the two researchers were resolved in the presence of a supervisor, facilitating the attainment of consensus on study inclusion and data extraction.

### Search strategy

**Fig. 1:**
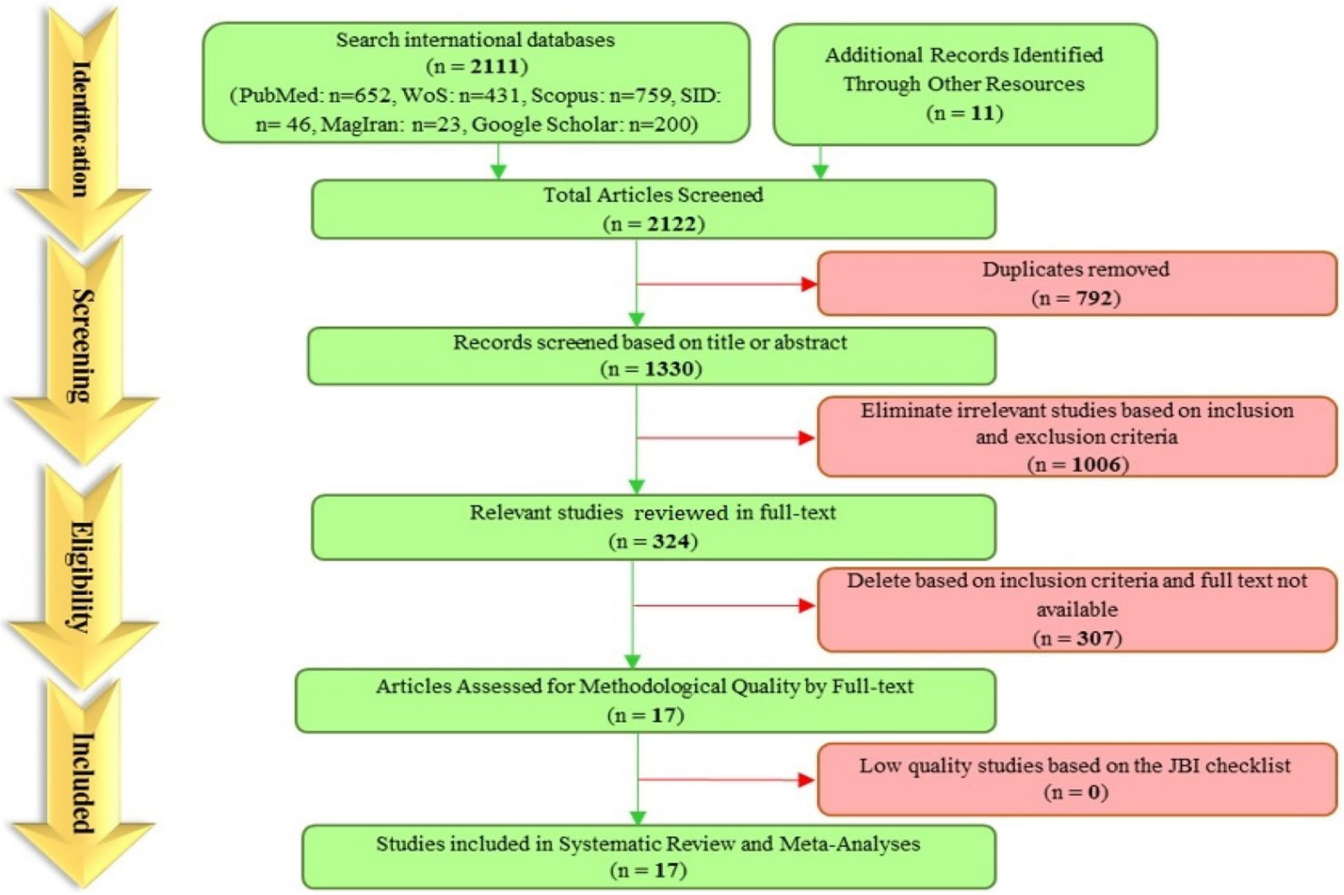
A flowchart diagram of the study search and selection

To find relevant studies to answer the question “What is the prevalence of sexual disorders in cardiovascular patients?”, Persian information sources (https://www.sid.ir) [19] Scientific Information Database and MagIran (https://www.magiran.com) and the international databases of PubMed, Scopus and (WoS) Web of Science were searched. To determine the search strategy in different databases, relevant and validated keywords were used using (MeSH) Medical Subject Headings for PubMed and combined using OR and AND operators. For example, the PubMed search strategy was determined as follows:

(((Prevalence[MeSH Terms]) OR (Prevalence*[Title/Abstract])) AND ((((((((((((((((((((((((((("sexual dysfunctions, psychological"[MeSH Terms]) OR ("Dysfunction, Psychological Sexual"[Title/Abstract])) OR ("Dysfunctions, Psychological Sexual"[Title/Abstract])) OR ("Psychological Sexual Dysfunction"[Title/Abstract])) OR ("Psychological Sexual Dysfunctions"[Title/Abstract])) OR ("Sexual Dysfunction, Psychological"[Title/Abstract])) OR ("Psychosexual Dysfunctions"[Title/Abstract])) OR ("Dysfunction, Psychosexual"[Title/Abstract])) OR ("Dysfunctions, Psychosexual"[Title/Abstract])) OR ("Psychosexual Dysfunction"[Title/Abstract])) OR ("Psychosexual Disorders"[Title/Abstract])) OR ("Disorder, Psychosexual"[Title/Abstract])) OR ("Disorders, Psychosexual"[Title/Abstract])) OR ("Psychosexual Disorder"[Title/Abstract])) OR ("Hypoactive Sexual Desire Disorder"[Title/Abstract])) OR ("Sexual Aversion Disorder"[Title/Abstract])) OR ("Aversion Disorders, Sexual"[Title/Abstract])) OR ("Disorders, Sexual Aversion"[Title/Abstract])) OR ("Sexual Aversion Disorders"[Title/Abstract])) OR ("Orgasmic Disorder"[Title/Abstract])) OR ("Disorders, Orgasmic"[Title/Abstract])) OR ("Orgasmic Disorders"[Title/Abstract])) OR ("Sexual Arousal Disorder"[Title/Abstract])) OR ("Arousal Disorders, Sexual"[Title/Abstract])) OR ("Disorders, Sexual Arousal"[Title/Abstract])) OR ("Sexual Arousal Disorders"[Title/Abstract])) OR (Frigidity[Title/Abstract]))) AND (((((((((((((((((((((((((((Cardiac*[Title/Abstract]) OR (Tricuspid*[Title/Abstract])) OR (Mitral*[Title/Abstract])) OR (Coronary[Title/Abstract])) OR (Coronaries[Title/Abstract])) OR ("Heart Failure"[Title/Abstract])) OR ("Heart Failure"[MeSH Terms])) OR (Heart[MeSH Terms])) OR (Heart*[Title/Abstract])) OR (angina*[Title/Abstract])) OR ("Angina Pectoris"[MeSH Terms])) OR (hypertensive[Title/Abstract])) OR (Hypertension*[Title/Abstract])) OR (Hypertension[MeSH Terms])) OR (Hypertense[Title/Abstract])) OR (Acute Coronary Syndrome[MeSH Terms])) OR (ACS[Title/Abstract])) OR ("Acute Coronary Syndrome"[Title/Abstract])) OR ("Myocardial Infarction"[Title/Abstract])) OR ("Myocardial Infarction"[MeSH Terms])) OR (MI[Title/Abstract])) OR ("Coronary Artery Bypass Surgery"[Title/Abstract])) OR (CABG[Title/Abstract])) OR ("Coronary Artery Bypass"[Title/Abstract])) OR ("Coronary Artery Bypass"[MeSH Terms])) OR (Cardiovascular*[Title/Abstract])) OR ("Cardiovascular System"[MeSH Terms])) (Fig. 1).

In the search process, no time and language limit was set so that all possible relevant studies be retrieved by January 14, 2023. Finally, to maximize the comprehensiveness of the search, Google Scholar and relevant sources were searched manually.

#### Inclusion criteria

1. Original research papers
2. Observational studies (cross-sectional, cohort, etc.)
3. Available full text
4. Studies reporting the percentage or frequency of sexual disorders in cardiovascular patients

#### Exclusion criteria

1. Studies irrelevant to the research question
2. Interventional studies (clinical trial, field trial, and social trial), qualitative research, case series, case reports, letters to the editor, articles presented at conferences, secondary studies, theses, and animal-based studies
3. Unavailability of the full text of the article after three emails to the corresponding author
4. Duplicates in different databases

### Selection process of studies

After selecting the search strategy for each database, all studies obtained from different databases were added to EndNote X8. First, all duplicates in different databases were removed. Then, the names of authors, institutions, and journals of all studies were removed. In the next step, the title and abstract were examined, and the irrelevant studies were excluded. Finally, the full text of the remaining papers was carefully examined by the inclusion and exclusion criteria, and the irrelevant studies were excluded. Finally, the papers that met all the predetermined inclusion criteria progressed to the qualitative evaluation stage.

### Qualitative evaluation of studies

The qualitative evaluation of the studies included in the meta-analysis was conducted using the Joanna Briggs Institute (JBI) checklist [38], which includes nine different dimensions of evaluation. These dimensions encompassed distinct aspects, such as sample frame, participants, sample size, study subjects and the setting described in detail, data analysis, valid methods of identifying conditions, measuring the situation, statistical analysis, and adequate response rate. The responses included yes, no, or not applicable (NA). The minimum and maximum scores based on JBI criteria were 0 and 9, respectively. A score of 1–3 represented low quality, a score of 4–6 showed medium quality, and a score of 7–9 was considered high quality [39].

### Data extraction

To extract data, a ready-made checklist was used, which enquired about the first author’s name, year of publication, country and continent of study, sample size, age, type of study, diagnostic tool, prevalence percentage, research population, and qualitative evaluation score with JBI used.

### Statistical analysis

The dependent variable investigated in this study was the percentage of sexual disorders in CVD patients. The results of different studies reporting the percentage or relative frequency of sexual disorders were integrated. The heterogeneity of studies was checked using *I*^2^. A value below 50% indicates low heterogeneity, while a value exceeding 50% suggests high heterogeneity. As the heterogeneity of the results of studies included in the meta-analysis was found to be high (*I*^2^ ˃ 50%), a random effects model was used. In this model, parameter changes among studies were also calculated to make the results of this model more generalizable than the fixed effect model when the heterogeneity is high [40]. Sensitivity analysis was used to identify the source of heterogeneity. To check publication bias, Begg and Mazumdar rank correlation and Egger’s test were used. Meta-regression was also run to investigate the relationship between the global prevalence of sexual disorders in cardiovascular patients with sample size, year of publication, age, and JBI score. The data were statistically analyzed using Comprehensive Meta-Analysis (Version 2). A *p*-value of less than 0.05 was considered statistically significant.

### Ethical approval

This study is the result of research project no. 4010048 approved by the Student Research Committee of Kermanshah University of Medical Sciences. Ethics approval was received from the ethics committee of deputy of research and technology, Kermanshah University of Medical Sciences (IR.KUMS.REC.1402.107).

## Results

### Study selection

In the systematic search, a total of 2122 potentially relevant studies were identified from the selected databases, and 792 duplicates were excluded. After evaluating the title and abstracts, 1006 studies were excluded, and 324 were evaluated in full text, of which 307 studies did not meet the inclusion criteria and were excluded. Finally, a total of 17 relevant studies were deemed suitable for inclusion in the meta-analysis. The study selection process is visually depicted in Fig. 1, which illustrates the flow of studies in accordance with the PRISMA guidelines.

### General characteristics of studies

The selected studies in this systematic review and meta-analysis were conducted between 2003 and 2021. The most frequent publications (*n* = 3) originated from studies conducted in Israel. Among the included studies, the research conducted by Herut et al. [41] reported the largest sample size with 19,131 participants, while the study by Schwarz et al. [42] had the smallest sample size of 100 participants. The highest quality score based on the JBI checklist belonged to the studies conducted by Rinkūnienė et al. [23], Rusiecki et al. [43], Fafiolu et al. [44], Mulat et al. [45], and Schwarz et al. [42]. The characteristics of the studies included in the systematic review and meta-analysis are provided in Table 1.

**Table 1:** Summary of studies included in systematic review and meta-analysis

First author, year, (references)	Country (continent)	Age (years)	Mean (age)	Place of study	Sample size			Prevalence %	Diagnostic tool	Population	Type of study	Quality score
					Total	Female	Male					
Fafiolu et al., 2014 [46]	Nigeria (Africa)	2014	50	South-West Nigeria	202	–	202	75%	International Index of Sexual Health Inventory for Men	Hypertensive	Cross-sectional	9
Koh, 2013 [47]	Malaysia (Asia)	2013	60.5	Malaysia	510	-	510	90%	International Index of Erectile Function	Ischemic heart disease	Cross-sectional	8
Hebert et al., 2008 [48]	USA (America)	2010	66	Tbilisi, Georgia, Eastern Europe	290	93	197	61.7%	Sexual Health Inventory for Men [1]	Heart failure	Cross-sectional	8
Oshodio et al., 2010 [49]	Nigerian (Africa)	2010	52	Nigeria	186	107	79	56/7%	The Arizona Sexual Experiences Scales (ASES) and General Health Questionnaire (GHQ-12)	Hypertension patients	Cross-sectional	7
Heikkilä et al., 2017 [50]	Finland)Europe)	2017		Southwestern Finland	665	-	665	52%	IIEF-5 score International Index of Erectile Function	Diastolic blood pressure (DBP)	Cross-sectional	7
Heruti et al., 2008 [51]	Israel (Asia)	2007	25–55	Israel	19,131	-		25%	SHIM questionnaire	Cardiovascular risk factors	Cross-sectional	8
Hood and Robertson, 2004 [52]	Paisley)Europe)	2004		Scotland	150	-		61%	International Index of Erectile Function	Cardiac rehabilitation	Cross-sectional	6
Mittawae et al., 2006 [53]	Egyptian (Africa)	2006	59.2	Egyptian	800	-	800	43/2%	International Index of Erectile Function	Hypertensive population	Cross-sectional	8
Montorsi et al., 2003 [54]	Italy)Europe)	2003	62.5	Switzerland	300	-	300	49%	International Index of Erectile Function	Patients with acute chest pain and angiographic ally	Cross-sectional	8
Mulat et al., 2010 [45]	Israel (Asia)	2010	67	Israel	242	-	242	76%	Sexual Health Inventory for Men [1] and the Mental Health Inventory-5 (MHI5) questionnaires	Coronary artery disease	Cross-sectional	9
Neiman et al., 2017 [55]	USA (America)	2017	31.9	USA	105	52	53	24%	Sexual Health Inventory for Men or Female Sexual Function Index	Adults with congenital heart disease (CHD)	Cross-sectional	7
Ramírez et al., 2016 [56]	Spain)Europe)	2016	53.7	Spain	440	-		42%	International Index of Erectile Function	Patients with cardiovascular risk factors	Cross-sectional	7
Rinkūnienė et al., 2021 [57]	Lithuania (Europe)	2021	57.6	Lithuania	171	-		62%	International Index of Erectile Function (IIEF-5)	Patients after a myocardial infarction	Cross-sectional	9
Roth et al., 2003 [58]	Israel (Asia)	2003	60	Israel	1412	-		57%	Utilizing IIEF-15, a 15-item multidimensional, self-administered questionnaire	Patients with diabetes, hypertension, or both diseases	Cross-sectional	8
Rusiecki et al., 2020 [43]	Poland)Europe)	2020	65	Poland	731	-		93.0%	IIEF-5 score	Patients with coronary artery disease	Cross-sectional	9
Schwarz et al., 2008 [42]	USA (America)	2010	59	USA	100	24	76	85%	International Index of Erectile Dysfunction (IIEF-5) questionnaire (2), the semi-structured interview on erectile dysfunction questionnaire, the Index of Sexual Life questionnaire, and the Female Sexual Function Index for determination of female sexual dysfunction	Chronic heart failure	Cross-sectional	9
Son et al., 2016 [59]	Korea (Asia)	2016	60	Korean	161	-	161	72.2%	International Index of Erectile Function (IIEF-5)	Coronary artery disease (CAD)	Cross-sectional	8

### Meta-analysis of global prevalence of sexual disorders in cardiovascular patients

**Fig. 2:**
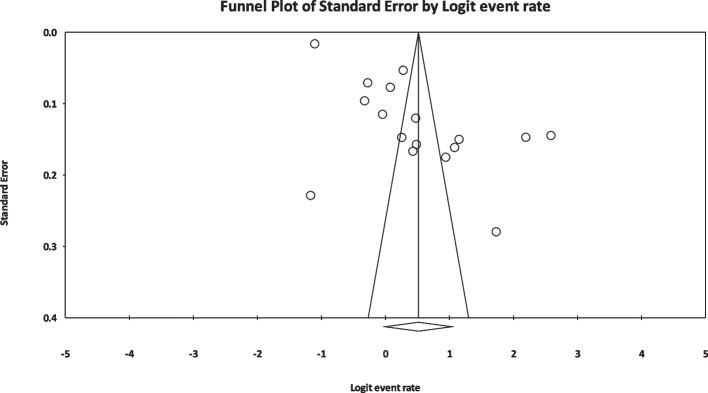
Funnel plot estimating the global prevalence of sexual disorders in cardiovascular patients based on the random effects model

**Fig. 3:**
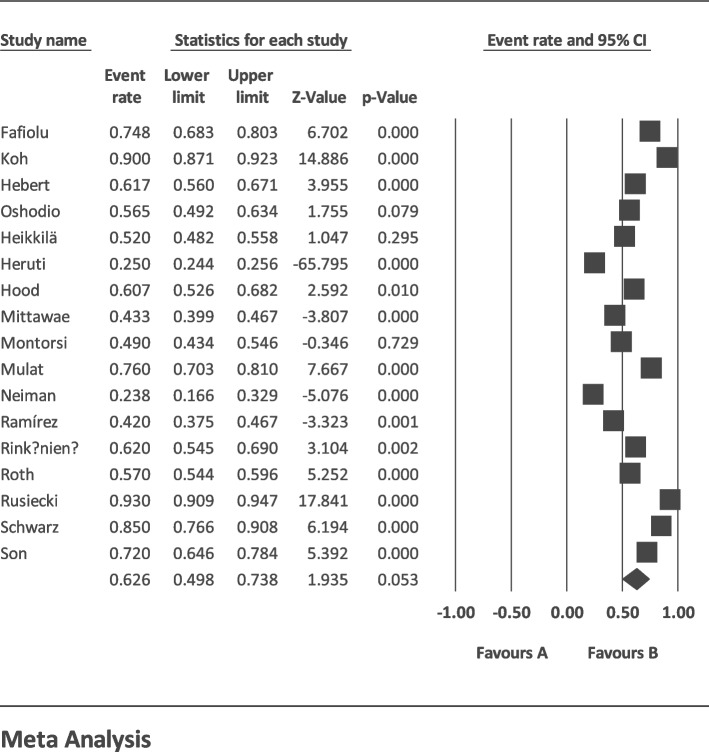
Forest plot of global prevalence of sexual disorders in cardiovascular patients based on random effects model

**Fig. 4:**
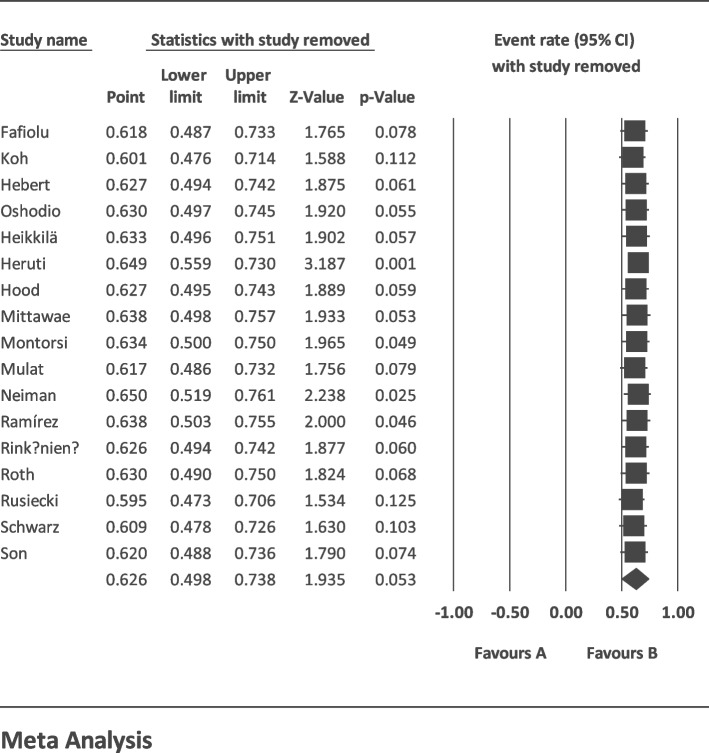
Sensitivity analysis diagram of global estimation of sexual disorders in cardiovascular patients

The I2 index for the global prevalence of sexual disorders in cardiovascular patients showed a great heterogeneity among the studies (*I*^2^ = 99.40%); thus, the data were analyzed using the random effects model. According to the results of Begg and Mazumdar’s rank correlation and Egger’s test, there was no publication bias in the studies at *p* < 0.1 (*p* = 0.901 and *p* = 0.456) (Fig. 2). Having integrated the results of the studies included in the meta-analysis, the global estimate of sexual disorders in cardiovascular patients was estimated at 62.6% (*CI* 95%: 49.8–73.8%) based on a random effects model. The black square is the percentage, and the length of the line segment is the 95% confidence interval in each study. The rhombus shows the global prevalence of sexual disorders in cardiovascular patients (Fig. 3). The results of the sensitivity analysis showed that the final result does not change significantly by removing any of the studies (Fig. 4). According to the GRADEpro software, the level of evidence was found to be very low (Table 2).

**Table 2:** Certainty of prevalence of sexual dysfunction in cardiovascular disease

Certainty assessment							Summary of findings		
Participants (studies) Follow-up	Risk of bias	Inconsistency	Indirectness	Imprecision	Publication bias	Overall certainty of evidence	Study event rates (%)		Impact
							With [comparison]	With pre	
0 (17 observational studies)	Not serious	Very serious^a^	Not serious	Not serious	None	⨁◯◯◯ Very low	The global estimate of sexual disorders in cardiovascular patients was estimated at 62.6% (*CI* 95%: 49.8–73.8%) based on a random effects model		

*CI* confidence interval

Explanations: ^a^I^2 index showed a great inconsistency

### Meta-regression

**Fig. 5:**
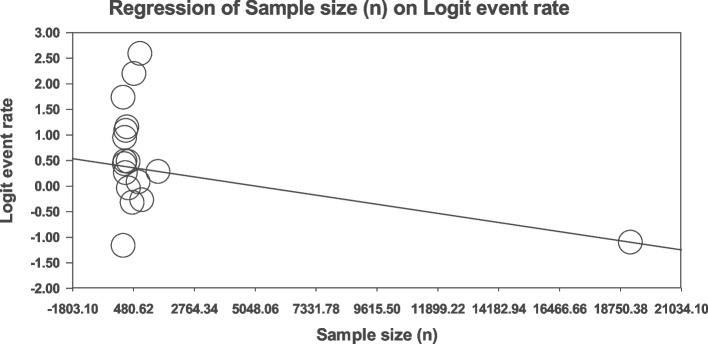
Meta-regression analysis of the relationship between sample size and global prevalence of sexual disorders in cardiovascular patients

**Fig. 6:**
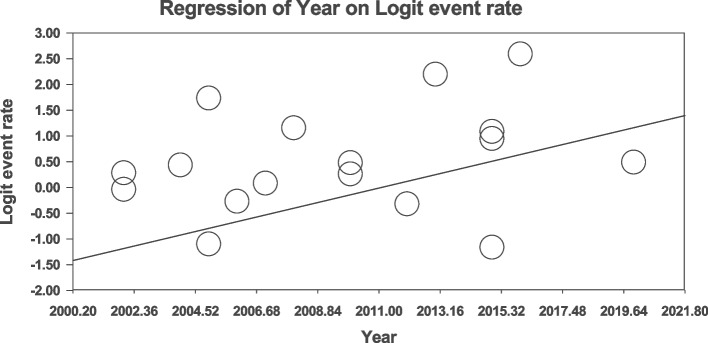
Meta-regression analysis of the relationship between the year of study and global prevalence of sexual disorders in cardiovascular patients

**Fig. 7:**
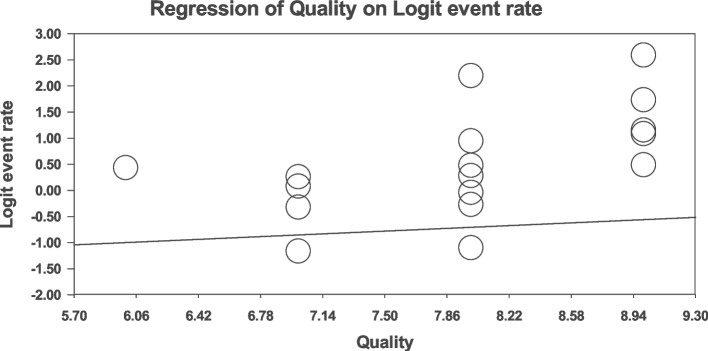
Meta-regression analysis of the relationship between JBI score and global prevalence of sexual disorders in cardiovascular patients

**Fig. 8:**
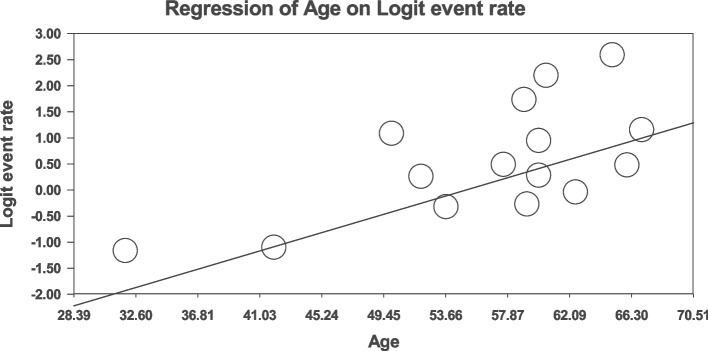
Meta-regression analysis of the relationship between age and global prevalence of sexual disorders in cardiovascular patients

Meta-regression analysis was used to test the relationship between sample size (Fig. 5), publication year (Fig. 6), and JBI score (Fig. 7) and age (Fig. 8) with the global prevalence of sexual disorders in cardiovascular patients. All these relationships were statistically significant in patients with CVDs (*p* < 0.01). With an increase in the sample size, the global prevalence of sexual disorders in cardiovascular patients decreased (Fig. 4). With an increase in the year of study, age and JBI score increased (Figs. 5, 6, 7 and 8).

## Discussion

CVDs can significantly impact in sexual activity by leading to challenges in both physical and mental aspects [11]. Scientific evidence also shows that SD is prevalent up to 42% in patients with CVDs [28, 29]. Although several seminal studies have been conducted worldwide in recent years to investigate the prevalence of sexual disorders among patients with CVDs, these studies have often been limited by their focus on specific geographic regions and small sample sizes, resulting in varying reported prevalence rates. Therefore, the present study aimed to address these limitations by conducting a comprehensive systematic review and meta-analysis, integrating global prevalence statistics, and synthesizing the contradictory findings from existing research on sexual disorders in patients with CVDs.

Having reviewed the studies retrieved from different databases, finally, 17 studies on the prevalence of sexual disorders were included according to the eligibility criteria. Having analyzed the results of studies, the global estimate of sexual disorders in cardiovascular patients was estimated at 62.6%. In a cross-sectional study of 171 patients, Rinkūnien et al. estimated the overall prevalence of erectile dysfunction after myocardial infarction at 62% [23]. The results of this study are similar to the results obtained in the present study. Rico Kano et al. showed that depression, anxiety, and monotonous and repetitive sexual relations significantly account for changes in sexual behavior in people, especially the elderly [60]. The existing literature also confirms that mental illness is strongly associated with sexual function [61]. As for the relationship between heart disease and the prevalence of mental disorders, the results of a meta-analysis showed that the prevalence of depression, anxiety, and stress in cardiovascular patients is high [62, 63]. In light of the results of the related literature, it can be concluded that the high prevalence of mental disorders such as depression, anxiety, and stress in cardiovascular patients plays a major role in sexual disorders. In agreement with the aforementioned argument, in a study by Krzastek et al., coronary artery disease and depression were introduced as two risk factors for changing sexual function [64].

Two additional studies conducted by Tirgari et al. [2] and Johansen et al. [15] reported the prevalence of sexual disorders in Iranian cardiovascular patients as 89% [2, 13], which is higher than the present finding. Although the results of these studies point to a high rate of sexual disorders in cardiovascular patients, the different statistical findings can be due to different methods, data collection instruments, the number of papers included, setting of study, and research populations.

The meta-regression analysis showed an increasing trend in the prevalence of sexual disorders with the increasing years, which does not signal improvement in health and sexual functioning of this population. This increasing trend requires investigation and management with the aim of preventing or controlling the aggravation of conditions by policy makers. Since sexual satisfaction is an important indicator of the quality of life and life satisfaction, the follow-up and treatment of sexual disorders should be attended more carefully.

The findings from the meta-regression analysis revealed a positive association between age and the prevalence of sexual disorders. One reason for this increasing trend may be the passage of life and entering the old age, which is associated with natural sexual cycle disorders compared to younger age. Age-related biological changes and the presence of regular challenges in sexual functioning are prevalent factors among patients as they grow older [25].

The results of a study in India provided insights into the sexual problems of the elderly population. This finding confirms that sexual problems are very common among elderly men and women. These findings also show that biology plays an important role in men’s sexual functioning, while psychology plays an important role in women’s sexual functioning [65]. Evidently, older patients have a lower level of education and find it harder to access information resources to identify sexual problems, follow-up, and treatment. In general, it seems that sexual health in old age is a neglected need and has been given significantly less attention [66]. The sexuality of the elderly and the related issues have been neglected not only from a therapeutic point of view but also from a research point of view, though it is of an utmost importance to health professionals [67, 68]. With advances in medicine, life expectancy has increased too. Therefore, addressing sexual issues in the elderly population with and without heart disease becomes increasingly important. Although the results of this study are important, there is a fundamental point. The current study is the generalizability of the study results due to the high sample size of one of the studies in Israel (75%) that the comparative weight of the studies heavily leans on. In the meta-analysis, larger studies carry more weight in determining the overall results. The percentage study weights indicate the contribution made by each study. However, it is important to acknowledge that the findings may not be equally applicable to different populations. Factors such as the selection of the sample, cultural differences, and environmental factors can impact the generalizability of the results. To ensure a broader applicability, scientists must conduct studies that represent the larger population. Taking these factors into consideration is essential when interpreting and applying the findings of the present study. Finaly and with all things considered, the high and significant prevalence of sexual disorders in cardiovascular patients in the present systematic review and meta-analysis shows the need for further investigation and follow-up for the disease. In addition, the evidence showed very low certainty, so we should interpret the results with caution. Most of the studies included in the meta-analysis assessed sexual dysfunction using self-reported data. While these measures provide valuable insights, they also bring about subjectivity biases that must be carefully considered. Self-reported measures are inherently subjective because they rely on individuals’ perceptions and interpretations of their own experiences. When it comes to sexual dysfunction, this subjectivity can be further compounded by the sensitive and intimate nature of the topic. As a result, individuals may underreport or overreport their symptoms based on a variety of personal and social factors. It is crucial for researchers and healthcare professionals to recognize that self-reported measures are just one part of a comprehensive assessment of sexual dysfunction. By integrating multiple sources of information, including self-reports, partner reports, and clinical evaluations, a more nuanced and accurate understanding of an individual’s sexual function can be achieved [69, 70]. It is recommended that future studies incorporate various methods to assess sexual dysfunction such as multimodal method. Multimodal assessment is an approach that involves using multiple methods or modes of assessment to gather information. It goes beyond traditional paper-based tests and includes various forms of assessment such as observations, interviews, and performance tasks. Sexual disorders are significant sexual health problems faced by cardiovascular patients, which can lead to dissatisfaction with life and a low quality of life. Establishing policies and strategies to control risk factors based on valid information can reduce the health-related and economic burden that these disorders place on cardiovascular patients in the long run. Recognizing populations at higher risk and providing effective, high-quality regular medical care can slow the progression of the disease and reduce complications [71].

The review showed that not all studies reported the prevalence in men and women separately to perform subgroup analysis. The high heterogeneity in the studies (more than 99%) led us to perform a meta-regression analysis to assess the effect of the underlying factors. Sexual functioning is a complex and multifaceted aspect of human life that can be influenced by a wide range of factors. While studies examining the relationship between certain factors and sexual functioning have been conducted, it is important to recognize that there are numerous other variables that can significantly impact an individual’s sexual health. One of the key factors that can impact sexual functioning is underlying health conditions. These conditions can lead to physical symptoms such as fatigue, pain, and reduced libido, all of which can contribute to sexual dysfunction. Moreover, the psychological burden of living with a chronic illness can also take a toll on one’s sexual well-being. Another important factor that is often overlooked is the use of medications, particularly those prescribed for cardiovascular conditions. Many medications, such as beta-blockers and certain antidepressants, can have side effects that impact sexual functioning. These can include reduced libido, erectile dysfunction, and difficulties achieving orgasm. Unfortunately, these side effects are often not openly discussed with patients, leading to a lack of awareness and potential reluctance to seek help. Mental health, including conditions such as anxiety, depression, and stress, can also have a profound impact on sexual functioning. These conditions can diminish libido, disrupt arousal and orgasm, and create barriers to intimacy. Furthermore, the stigma surrounding mental health can make it difficult for individuals to seek help and support for their sexual concerns. As researchers delve deeper into understanding the intricate relationship between cardiovascular health and sexual well-being, it becomes increasingly evident that an array of variables play a pivotal role in shaping this relationship. To truly comprehend the underlying mechanisms and potential interventions, future research must prioritize the collection of detailed information and robust analyses of these variables [72, 73]. Alos, it is suggested that further systematic reviews and meta-analyses explore the prevalence of sexual disorders in cardiovascular patients as well as the prevalence of sexual disorders in other populations such as diabetic patients, hemodialysis patients, and cancer patients.

The present systematic review revealed that the design of the included studies was cross-sectional. Cross-sectional studies are valuable in providing a snapshot of the relationship between CVD and sexual dysfunction at a specific point in time. However, they are inherently limited in their ability to establish cause and effect. This is mainly because they do not follow individuals over time, making it difficult to determine the temporal sequence of events. The cross-sectional study design, often employed to investigate this correlation, imposes limitations on our ability to definitively establish causality or directionality. When it comes to understanding the intricate connections between sexual dysfunction and cardiovascular disease, employing longitudinal cohort studies is essential. These studies follow subjects over extended periods of time, allowing for the analysis of potential causal pathways and the confirmation of the temporality of associations. By delving into this research, we can gain insight into whether sexual dysfunction acts as an early marker or risk factor for cardiovascular disease or if it arises as a complication or consequence of cardiovascular pathology.

Based on the study results, the limited number of studies on the prevalence of sexual disorders in cardiovascular patients in some continents is discussing. Cardiovascular disease (CVD) is a leading cause of mortality worldwide, with significant variations in prevalence across different regions and populations. However, the limited number of studies from certain world regions where CVD rates are high indicates reduced generalizability of the global prevalence estimates. By expanding the geographical scope of cardiovascular disease studies, we can gain a more comprehensive understanding of the global burden of CVD, develop tailored interventions for specific populations, and promote equity in global health research [74]. In addition, the text highlights a limitation in some research papers due to nonrandom sampling methods used, which could introduce biases in sample characteristics and responses. The use of probability sampling is suggested as a solution to enhance sample representativeness and reduce sampling errors. Also, the review did not consider publication biases from unpublished studies, gray literature, or non-English papers. Including these sources could provide more data on prevalence rates.

## Conclusions

The results of the present study show that the prevalence of sexual disorders in cardiovascular patients is high. Therefore, it is recommended that health policymakers pay closer attention to the prevention and control of these disorders. Cardiologists should consider the side effects of cardiac medication on the sexual functioning of patients when prescribing medication. By acknowledging the impact that these medication can have on sexual health, healthcare professionals can take proactive measures to minimize any adverse effects. Patients should receive necessary information regarding the potential impact of prescribed medications on sexual function, allowing them to make informed decisions about their treatment options. Given the significant impact that sexual health has on overall well-being, mental health and quality of life, addressing this issue should be a priority in the healthcare system.

## Data Availability

The datasets used in the study are available from the corresponding author on reasonable request.

## Abbreviations

GP: Global prevalence
SD: Sexual dysfunction
CP: Cardiovascular patients
SRMA: Systematic review and meta-analysis
ED: Erectile dysfunction
CVD: Cardiovascular disease
